# Micropulse P3™ (MP3) Laser for Glaucoma: An Innovative Therapy

**DOI:** 10.5005/jp-journals-10008-1244

**Published:** 2018-08-01

**Authors:** Maria Cecilia Aquino, Dawn Lim, Paul TK Chew

**Affiliations:** 1 Ophthalmologist, Consultant Ophthalmologist, Associate Professor, National University Hospital, National University Health System, Singapore; 2 Associate Professor, National University of Singapore, National University Health System, 5 Lower Kent Ridge Road, Singapore

## Abstract

**How to cite this article:** Aquino MC, Lim D, Chew PTK. Micropulse P3™(MP3) Laser for Glaucoma: An Innovative Therapy. J Curr Glaucoma Pract 2018;12(2):51-52.

Laser therapy remains a useful, noninvasive option in the widening array of glaucoma treatment modalities.Traditional ciliary body lasers are generally cyclodestructive and concern about its use stem from the risks of sight threatening sequelae. Transcleral diode laser cyclophotocoagulation (TSCPC) (Fig. 1) is a treatment modality for various types of glaucoma refractory to other forms of management. It is presently the preferred method of ciliary body ablation due to its ease of application and noninvasive method. Despite these, it tends to be reserved for eyes with little or poor visual prognosis and regarded as a treatment of last resort. This practice has persisted, because of the unpredictable effects of the laser, limited success, need for multiple treatment and ocular complications, such as hypotony, phthisis bulbi, hyphema, inflammation, macular edema, choroidal/retinal detachment, vitreous hemorrhage to name a few.

**Fig. 1:**
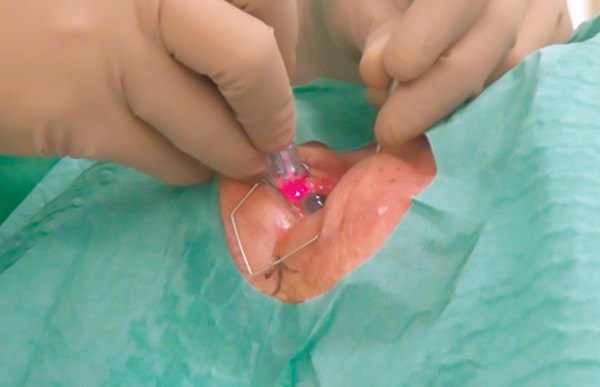
Transcleral diode laser cyclophotocoagulation.

## HOW DOES MP3 WORK?

**Fig. 2:**
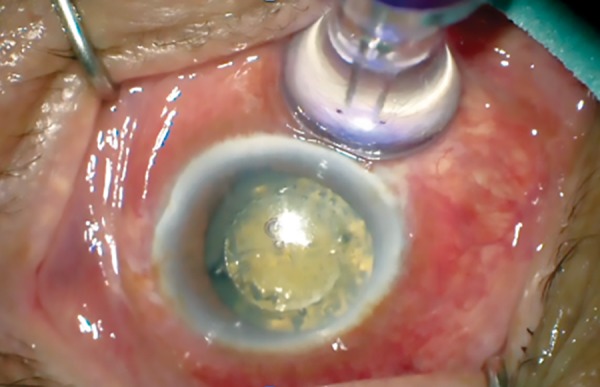
Micropulse novel probe design (MP3) application

A new form of ciliary body laser termed micropulse P3™ (MP3) using 810 nm infrared diode has been developed. It uses a novel probe design (micropulse P3™ glaucoma device) (Fig. 2) of a ball-lens tip contact probe that houses a quartz fiberoptic cable 600 μm in diameter, with its hemispherical tip protruding 0.7 mm from the handpiece to facilitate accurate positioning of the fiberoptic tip 3 mm posterior to the limbus to target the pars plana. The probe is moved in a painting manner circumferentially in a continuous fashion avoiding the 3 and 9 o’clock meridians. Micropulse laser application refers to delivering a series of repetitive short pulses of energy with rest periods inbetween. This technique theoretically minimizes collateral tissue damage and hence avoids pronounced tissue disruption of ciliary body seen in histological specimens after conventional continuous wave cyclophotocoagulation.^1^ It is hypothesized that this form of ciliary body treatment lowers intraocular pressure (IOP) by enhancement of existing uveoscleral outflow channels without photocoagulating the target site.

## HOW EFFECTIVE AND SAFE?

Micropulse technology in glaucoma therapy has paved the way for an efficient and safe procedure applicable to all glaucoma types. The pioneering work and experience of Chew et al. both in the pilot^2^ and randomized^3^ studies on advanced, refractory glaucomas have provided evidence that MP3 lowers IOP by an average of 45% from baseline with minimal to nil complications without any incidence of hypotony and phthisis bulbi after 18 months of follow-up. With the micropulse laser machine set at 31.3% duty cycle (2 watt power, on time 0.5 msec and off time 1.1 msec for the total exposure of 100 seconds) the desired effect is reached. Reports on MP3 by other investigators adapting the technology demonstrated the same efficacy. Williams, Moster et al.^4^ treated patients with refractory glaucoma and reported up to 51% IOP reduction at the last follow-up (7.8 months ± 4.5). Maslin and Noecker^5^ reported their experience after 12 months of follow-up and found an average of 41.6% IOP reduction without any hypotony. Radcliffe et al.^6^ observed a mean IOP reduction of 29.8% after 3 months. To show the non destructive effects of MP3 on the ciliary body, Shan Lin et al.^7^ performed ultrasound biomicroscopic imaging before and after treatment. No ultrasonographic evidence of morphological changes, destruction of adjacent structures nor suprachoroidal effusion were seen following the using of MP3. With encouraging results in lowering IOP, proven safety in advanced and medically refractory glaucoma, MP3 use and its clinical applications continue to be widen.

## WHAT IS ON THE HORIZON?

MP3 is increasingly used by eye surgeons in different centers worldwide. Modifications to the power, treatment duration, duty cycle or a combination of the three parameters have been described in the literature.^8,9^ New treatment protocols are being evaluated to address cases with minimal or no response to initial MP3. Its role as a potential replacement or adjunct to medical therapy is currently being evaluated. MP3 is now being increasingly used in combination with cataract surgery. MP3 as a temporizing therapy to lower IOP prior to glaucoma filtering surgery in medically refractory cases increases the safety and reduces the risks of devastating sight threatening complications (such as hypotonous maculopathy) before definitive glaucoma filtering surgeries especially in eyes with extremely high IOP preoperatively. Like all new technology, the expanding indications of MP3 remain to be explored.
